# Recent advances in adipose-derived mesenchymal stem cell-derived exosomes for regulating macrophage polarization

**DOI:** 10.3389/fimmu.2025.1525466

**Published:** 2025-02-03

**Authors:** Zhewei Dong, Yingli Fu, Zhongming Cai, Hao Dai, Yucang He

**Affiliations:** ^1^ Renji College, Wenzhou Medical University, Wenzhou, Zhejiang, China; ^2^ Department of Plastic Surgery, First Affiliated Hospital of Wenzhou Medical University, Wenzhou, Zhejiang, China; ^3^ Department of Breast Plastic Surgery, Plastic Surgery Hospital, Chinese Academy of Medical Sciences and Peking Union Medical College, Beijing, China

**Keywords:** exosomes, adipose-derived mesenchymal stem cells, macrophage polarization, M2 macrophages, inflammatory diseases

## Abstract

Adipose-derived mesenchymal stem cells (ADSCs) exhibit superior immunomodulatory properties and have broad therapeutic applications. They induce macrophage M2 polarization for anti-inflammatory responses. Exosomes derived from ADSCs (ADSC-EXOs) exhibit biological functions similar to those of ADSCs but can circumvent the limitations associated with cellular injection therapies. Potent anti-inflammatory substances contained in exosomes include the glycoprotein MFGE8, the cytokines such as prostaglandin E2, IL-6, and IGF, as well as non-coding nucleotides (miR-451a, miR-23, miR-30d-5p, let-7, lncRNA DLEU2, circRps5, Circ-Ptpn4, and mmu_ circ_0001359). The anti-inflammatory and immunomodulatory properties of these exosomes provide new perspectives for therapeutic approaches for graft inflammation, bone healing, acute lung injury, kidney stones, myocardial infarction, and diabetes-related diseases. This review summarizes the contents and functions of ADSC-EXOs, outlines their properties and the characteristics of macrophage phenotypes, and emphasizes their impact on macrophage polarization and their contribution to immune-related diseases.

## 1 Introduction

Mesenchymal stem cells (MSCs) have substantial medical and biological value and have become a research hotspot in biomedicine due to their excellent immunomodulatory properties and wide range of applications (1). MSCs are multi-differentiated cells derived from various tissues, including bone marrow, umbilical cord blood, and adipose tissue (2). Among these, adipose-derived mesenchymal stem cells (ADSCs) are easily accessible, minimally invasive, and easy to culture, with substantial medical promise and development potential (3). Exosomes possess great potential for cell-free therapies as key mediators of intercellular communication (4, 5). ADSC-derived exosomes (ADSC-EXOs) play vital role in regulating macrophage M1/M2 polarization, mediating inflammatory responses, and modulating immune functions (6). The M1 and M2 macrophage phenotypes represent two extremes of activation states crucial to both the progression and recovery of inflammation in the body (7). M1 macrophages, representing the classically activated phenotype, contribute to tissue damage by releasing a wide range of cytokines and chemokines that trigger pro-inflammatory, anti-microbial, and tumorigenic activities. In contrast, M2 macrophages, which have an alternatively activated phenotype, exert anti-inflammatory, tissue regeneration and repair, angiogenic, and immunomodulatory effects (8). This article reviews the role of ADSC EXO in regulating macrophage M2 polarization and in the treatment of diseases such as bone healing, acute lung injury, kidney stones, fat graft survival and myocardial infarction (MI), which have been studied in recent years.

## 2 ADSC-EXOs

Extracellular vesicles are classified into different subtypes based on their diameter: exosomes (30–100 nm), microvesicles (100–1,000 nm), and apoptotic vesicles (1–5 μm) (9). Exosomes are important components of MSC secretion. MSC-derived exosomes are readily distinguishable by the presence of markers and proteins, including surface markers such as CD9, CD63, and CD81 of the tetraspanin family;, heat shock proteins (HSP60, HSP70, and HSP90), multivesicular bodies, biologically derived proteins (Alix and tumor susceptibility gene 101 [TSG101]), lipid-associated proteins, and phospholipases (10, 11). Notably, the phenotype and biological effects of exosomes may change depending on the type of MSCs source (12). MiRNAs are one of the major components of exosomes that are protected from RNAase attack by an exosomal lipid bilayer outside of the exosome (4). Among them, miR-155 and miR-146 are involved in physiological and pathological processes such as organism development, epigenetic regulation, and immune regulation, and miR-23b, miR-451, miR-223, miR-24, miR-125b, miR-31, miR-214, and miR-122 are involved in tumorigenesis and tumor progression (1).

ADSC-EXOs possess numerous medicinal and biological applications. They possess the advantages of being small in size, the ability to penetrate biological membranes (capillaries and the blood-brain barrier), low immunogenicity, and ease of storage (11). Currently available or developing separation techniques include ultracentrifugation-based separation, size-based techniques, precipitation techniques, immunoaffinity capture, and combinations of these techniques (4). Exosome production is simple and efficient, and they can be extracted from culture medium using approaches such as ultracentrifugation or produced on a large scale using specialized cell lines (13). Exosomes are easy to store, structurally stable, straightforward, unaffected by storage at -20°C for one week, and retain their activities during long-term storage at -80°C (14). Exosomes are safer, and in contrast their use avoids issues associated with MSC therapy, such as cell survival, regenerative capacity, immune rejection, and tumor differentiation (15). These factors provide a solid foundation for the commercial production of ADSC-EXOs and highlight their therapeutic value (16).

## 3 Macrophage polarization

Macrophages are important immune cells involved in infection prevention, tissue repair, angiogenesis, and immunomodulatory processes. They are also important contributors to the promotion and resolution of inflammation. Macrophages adopt two distinct functional phenotypes in response to different signals in various tissue microenvironments: classically activated macrophages (M1) and alternatively activated macrophages (M2) (17). Among these, M1 macrophages exhibit potent antimicrobial properties, high antigen-presenting capacity, and activate the Th1 response, leading to strong pro-inflammatory and antimicrobial effects, whereas M2 macrophages promote tissue repair and regeneration with an anti-inflammatory response relative to M1 (8).

Macrophage polarization and function are primarily regulated by a network of signaling molecules, transcription factors, epigenetic mechanisms, and post-transcriptional regulators (18). Typically activated by lipopolysaccharide (LPS) and Th1 cytokines (for example, IFN-γ and TNF-α), macrophages undergo M1 polarization, releasing various cytokines and chemokines (for example, TNF-α, IL-1α, IL-1β, IL-6, IL-12, CXCL9, and CXCL10), which then interact with unpolarized macrophages, creating a positive feedback loop (8, 9). The transcription factors studied and elucidated are the NF-κB (p65 subunit), STAT1, STAT5, IRF3, and IRF5. NF-κB and STAT1 are the two main transcription factors involved in M1 macrophage polarization (8). M2 polarization is controlled by downstream signals from cytokines such as IL-4, IL-13, IL-10, IL-33, and TGF-β (8, 19). Of these, cytokines (for example, IL-33 and IL-25) promote M2 activation by producing Th2 cytokines, and only IL-4 and IL-13 directly induce M2 activation (20). Key transcription factors regulating M2 gene expression include STAT6, IRF 4, JMJD 3, PPARδ, and PPARγ, and it is currently believed that the STAT6 pathway activates M2 macrophages (8). Two antagonistic pathways of arginine metabolism are responsible for the polarity of M1/M2 macrophages. M1 macrophages are associated with the iNOS pathway that uses arginine to produce citrulline and nitric oxide (NO), whereas M2 macrophages are associated with the arginase pathway that uses arginine to produce ornithine and urea (21).

M2 macrophages exert profound effects on tissue repair, cell growth, immune system regulation, inflammation, and apoptosis suppression. M2 macrophages can be divided into four subtypes: M2a, M2b, M2c, and M2d (8), each activated by different cytokines and transcription factors and displaying distinct secretions and effects. Among them, M2a macrophages are activated by IL-4 or IL-13, increasing the expression of IL-10, TGFβ, CCL17, CCL18, and CCL22, and enhancing endocytosis activity to promote cell growth and tissue repair (8). M2b macrophages are activated by immune complexes, Toll-like receptor (TLR) ligands, and IL-1β to release pro- and anti-inflammatory cytokines such as TNF-α, IL-1β, IL-6, and IL-10 to regulate the depth and breadth of the immune and inflammatory response (16).

## 4 How ADSC-EXOs regulate macrophage depolarization

**Figure 1:**
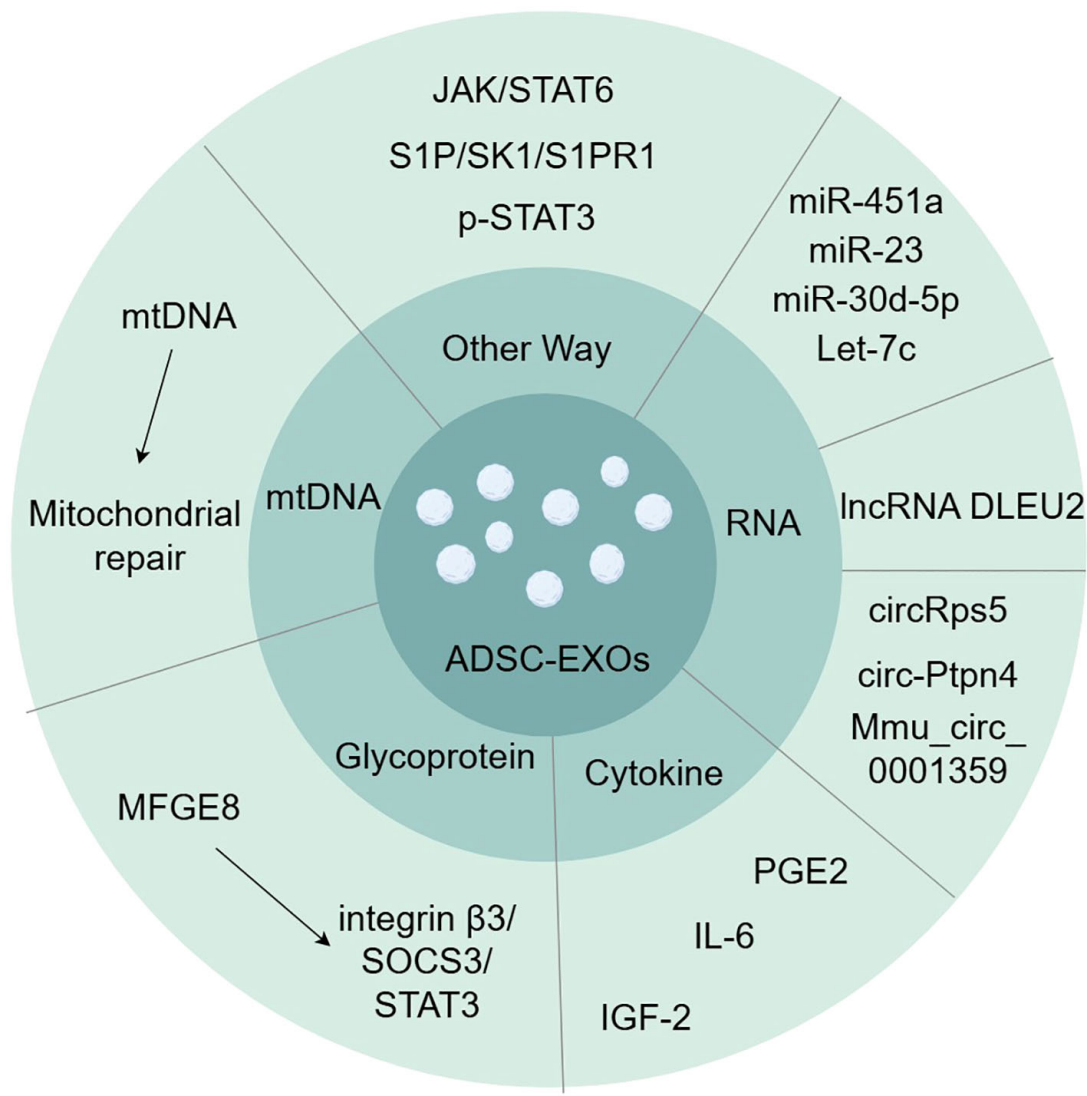
Exosomes contain various substances involved in inducing macrophage polarization, such as glycoprotein, cytokine, RNA, and mtDNA. (by Figdraw).

Exosomes are one way in which ADSCs regulate macrophage polarization in a cell-contact-free manner. Many signaling pathways are involved in macrophage polarization, including the PI3K/AKT, AK/STAT, NF-κB, Wnt/β-catenin, and Notch signaling pathways (6, 22). Experiments have identified several proteins, DNAs, mRNAs, and miRNAs in ADSC-EXOs that regulate the polarization and function of M1/M2 macrophages (**Figure 1**).

### 4.1 Glycoprotein in ADSC-EXOs

MFGE8 is a glycoprotein that promotes the clearance of dead or apoptotic cells and exerts anti-inflammatory effects by promoting the polarization of M2 macrophages (23, 24). ADSC-EXOs have been demonstrated to be rich in MFGE8 (25). Integrin β3 is one of the known MFGE8 receptors, and the signaling pathway for this receptor is integrin β3/SOCS3/STAT3 (23). Activation of this pathway increases STAT-3 phosphorylation, thereby mediating macrophage reprogramming toward M2 polarization (25).

### 4.2 Cytokines in ADSC-EXOs

Cytokines in ADSC-EXOs also induce M2 macrophage polarization. Prostaglandin E2 (PGE2) is a soluble and important immunomodulatory cytokine (26). Treatment with PGE2-enriched ADSC-EXOs resulted in a decrease in gene expression of M1-characterized cytokines (iNOS, IL-6, and TNF-α) and an increase in gene expression of M2-characterized cytokines (IL-10, Arg-1, and CD206), as well as a shift of macrophages from M1-type to M2-type in a rat model of colitis (27). IL-6 also mediates macrophage polarization in ADSC-EXOs (28). IL-6 exposure upregulates IL-4 receptor expression and responses in macrophages, leading to STAT6 phosphorylation, which, in turn, directs M2 macrophage polarization (29, 30). Insulin-like growth factor (IGF) is a serum component structurally similar to the insulin B chain (31). ADSC-secreted IGF-2 pre-programs maturing macrophages (31). The secretion of pro-inflammatory cytokines such as IL-12, IL-17, and IL-1β was reduced, and PD-L1 expression was upregulated in treated macrophages (31). IGF-2 exhibits a metabolic commitment to oxidative phosphorylation of macrophages (OXPHOS) and significantly alters the distribution of H3K27ac in macrophages, with significant reductions in the promoters and enhancers (e.g., Mir155) of key regulators involved in macrophage M1 activation and enhancements in a number of genes, such as the macrophage inflammation inhibitor methyl-CpG-binding protein 2 (Mecp2) (31–33).

### 4.3 RNA in ADSC-EXOs

miRNAs are a family of short non-coding nucleotides that regulate target genes at the post-transcriptional level and are important components of MSC exosomes that regulate cell growth and metabolism (34, 35). miR-451a is a highly expressed miRNA in ADSCs that specifically binds to the macrophage migration inhibitory factor (MIF) mRNA 3′-UTR, thereby reducing the expression of the downstream target MIF (3, 36, 37). MIF is an endocrine immune molecule that limits macrophage activity *in vivo*, is involved in immune regulation, and has been experimentally demonstrated to promote the polarization of M1 to M2 macrophages; however, the underlying mechanism has not yet been elucidated (3). Experiments suggest that the direct target of miR-23 in exosomes is interferon regulatory factor 1 (IRF1), and that miR-23 inhibits IRF1 to inhibit M1 macrophage polarization (38). Exosomal miR-30d-5p can target the 3′-UTR of Beclin-1 and Atg5 at the mRNA level, significantly inhibiting Beclin-1 and Atg5 expression and driving macrophage polarization from M1 to M2 (39). Let-7, the first miRNA identified, has been demonstrated to be a negative regulator of the pro-inflammatory response induced by TLR4 stimulation (40). Exosome-derived Let-7c significantly reduces the expression of the transcription factor CCAAT/enhancer-binding protein (C/EBP)-δ that plays a key role in the regulation of TLR4 in macrophages, thereby inhibiting M1 macrophage polarization (41).

Long non-coding RNAs (lncRNAs) are RNA molecules that are more than 200 nucleotides in length compared with miRNAs (42). They play critical roles in the regulation of cellular activity and behavior (42). ADSC-EXOs affect macrophage polarization by delivering lncRNA DLEU2 (42). It regulates mRNA expression by targeting miRNAs, and DLEU2 promotes macrophage M2 polarization by regulating the miR-106a-5p/LXN axis (42).

ADSC-EXOs also carry a non-coding circular RNA (circRNA) produced from a post-spliced exon, which is a naturally occurring family of non-coding RNAs highly expressed in the eukaryotic transcriptome (43). circRps5 possesses a stable circular structure that binds to miR-124-3p and reduces its levels, thereby inhibiting M1 macrophage polarization (44). ADSC-EXOs also deliver circ-Ptpn4 that downregulates the expression of miR-153-3p targeting the Nrf2 3′-UTR, resulting in enhanced Nrf2 expression and macrophage conversion from M1 to M2 (43). Mmu_circ_0001359 also links alternatively activated macrophages to the M2 phenotype by upregulating miR-183-5p expression, thereby promoting the expression of the transcription factor FoxO1 (45).

### 4.4 ADSC-EXOs restore mitochondria

In terms of mitochondria and mtDNA, ADSC-EXOs increased mitochondrial mtDNA levels and restored the levels of key molecules related to mitochondrial biosynthesis and homeostasis (PGC-1α, TFAM, and Sirt1) as well as key molecules related to the mitochondrial respiratory chain (cox-15, NDUFV2, and ATP5d) and mitochondrial membrane potential. OXPHOS activity and ATP production were increased, and macrophage mitochondrial reactive oxygen species (mROS) stress caused by LPS stimulation was alleviated, restoring oxidative phosphorylation process and mitochondrial function (46). Exosome-mediated blunting of ROS generated after oxidative stress in macrophage mitochondria promotes activation of inflammatory pathways such as NF-κB (47, 48). ADSC-EXOs switch macrophages from the M1 pro-inflammatory phenotype to the M2 polarized anti-inflammatory phenotype. Additionally, cells selectively package the mitochondrial components of exosomes, actively preventing the packaging of pro-inflammatory oxidized mitochondrial materials into exosomes, which may act as damage-associated molecular patterns (46).

### 4.5 Other ways

ADSC-EXOs significantly activated the JAK/STAT6 signaling pathway in macrophages (49). The JAK/STAT6 signaling pathway is a typical pathway involved in macrophage M2 polarization (49). When IL-4/IL-13 binds to receptors located on the cell membrane, JAK1 is phosphorylated, which immediately activates STAT6; this, in turn, activates M2-like genes such as YM1, Arg1, Fizz1, IL-10, and MGL1, ultimately initiating M2 macrophage polarization (49–51). Additionally, ADSC-EXOs activate the S1P/SK1/S1PR1 signaling pathway in macrophages, inhibit the expression of NF-κB p65 and TGF-β1, polarize macrophage M2, and suppress inflammatory responses (52). ADSC EXOs contain phosphorylated STAT3. Direct delivery of p-STAT3 to macrophages results in its binding to STAT3-targeted DNA and promotes Arg-1 promoter/enhancer transcriptional activation, thereby promoting M2 polarization (53).

## 5 Applications of ADSC-EXOs to regulate macrophage polarization

**Figure 2:**
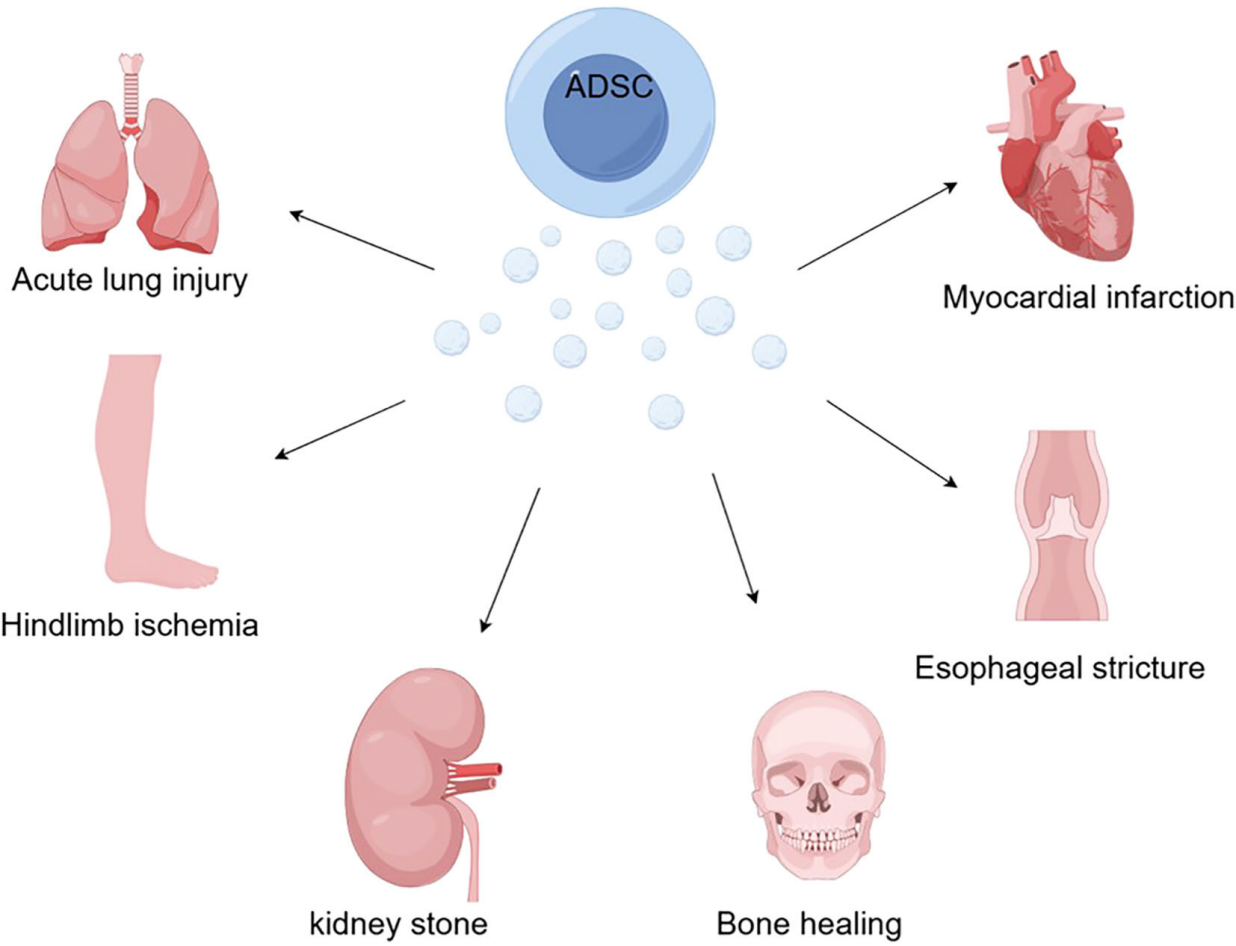
With the ability to induce macrophage polarization, mediate inflammatory responses, and regulate immunity, ADSCs offer new hope for bone healing, acute lung injury, esophageal stricture, kidney stones, myocardial infarction, and hindlimb ischemia caused by diabetes (by Figdraw).

Numerous successful ADSC-EXO therapy studies and technological explorations have been conducted in animal models over the past few years. This approach has been used experimentally with favorable results for graft inflammatory responses, bone healing, acute lung injury, esophageal stricture, kidney stones, myocardial infarction, and diabetes-derived diseases (**Figure 2**).

### 5.1 Acute lung injury

Acute lung injury (ALI) can be caused by acute pneumonia, sepsis, severe trauma, acute pancreatitis, and other causative factors (46). ALI is typically associated with extensive airway inflammation, hypoxemia, and tissue disorganization due to pulmonary immune abnormalities and altered vascular permeability during this period; however, it still exhibits a high mortality rate (35–55%) after treatment (e.g., improvement of mechanical ventilation) and poses a great threat to human health (54–56). LPS is a known predisposing factor that induces innate immune cells to secrete inflammatory mediators, thereby causing lung injury (57). This process is characterized by the collapse of alveolar structures, thickening of alveolar septa, changes in membrane transparency, and the infiltration of large numbers of inflammatory cells (46). In the experiment, mitochondrial function and immune homeostasis of lung macrophages in the LPS-induced ALI mouse model were improved under ADSC-EXOs treatment (46). ADSC-EXOs transfer mitochondrial components (especially mtDNA) to stressed lung macrophages, increase mitochondrial DNA levels, mitochondrial membrane potential, OXPHOS activity, and ATP production, and alleviate LPS-induced macrophage mROS stress thereby inhibiting TLR signaling activation and M1 macrophage polarization (46). In this process, decreased release of IL-1β, TNF-α, and iNOS, along with increased relative levels of anti-inflammatory cytokines such as IL-10 and Arg-1 attenuate the inflammatory response (46). This study provides a new approach to the treatment of LPS-induced ALI and raises the question of whether ADSC-EXOs can be effective in viral pneumonia, bacterial pneumonia, and autoimmune lung injury, and whether the efficacy of ADSC-EXOs can be improved using a form of nebulization.

### 5.2 Bone healing

Traumatic bone defects are typically associated with inflammation (3). The most commonly used clinical method, autologous bone grafting, has significant limitations, such as large defect areas and donor site discomfort (58, 59). However, allogeneic bone grafts can cause immune rejection and infection (60, 61). With the development of material technology, biomaterial implantation has attracted widespread attention as a potential solution. However, studies have reported that it can induce an inflammatory response that affects bone metabolism and new bone formation, leading to implant failure (3, 61, 62). Therefore, new solutions are urgently required to promote effective bone healing and regeneration. Recent experiments have indicated that the immune system cells are closely linked to the skeletal system cells and cooperate with each other (3). Bone defects due to trauma and tumors are typically accompanied by peripheral inflammation and immune dysregulation, including acute ischemia and hypoxia, the release of pro- and anti-inflammatory factors, and abnormalities in cellular metabolism (3). Thus, regulation of macrophage M1/M2 polarization with immunomodulatory effects is important for traumatic bone defects. A model of skull defects in rats was successfully established, new bone formation is promoted in cranial defect areas (3). ADSC EXO enriched with miR-451a inhibited the expression of MIF, promoted the shift of macrophages from pro-inflammatory to anti-inflammatory, and inhibited the expression of inflammatory factors such as NO, TNF-α, and IL-6, ultimately suppressing the inflammatory response related to bone defects and accelerated the bone healing in the experiment (3). The application of GNP hydrogels offers a new approach to bone healing; however, the specific mechanism of miR-451a enrichment in ADSC-Exos to promote the process of macrophage M1-to-M2 transition by downregulating the expression of MIF still needs to be investigated more deeply to guide subsequent clinical applications.

### 5.3 Kidney stones

Kidney stone formation, one of the most common urinary tract diseases, is closely associated with genetic, environmental, and metabolic factors (63). Kidney stones can be categorized into different types based on their chemical composition. Calcium oxalate (CaOx) stones are the most common and exhibit a high recurrence rate (70–80% in the last 20 years), posing a major threat to the urinary system (38). Several studies have demonstrated that inflammation-induced damage to the renal tubular epithelial cells alters the structure and polarity of the cell membrane surface, thereby promoting calcium oxalate crystal adhesion and stone formation (64). Macrophages and their M1/M2 polarization phenotypes are central to CaOx stone formation (38). The pathogenesis of CaOx crystals involves the promotion of M1-type macrophage polarization that damages renal tubular epithelial cells and promotes the development of CaOx crystal deposition. In contrast, M2-type macrophages phagocytose CaOx crystals, enhance anti-adhesion capacity, and protect renal tubular epithelial cells (65, 66). In the hyperoxaluria rat model, renal tubular injury scores are significantly decreased in the treatment group (38). IRF1 expression is inhibited by treatment with miR-23-enriched ADSC-EXOs, blocking the polarization of M1 macrophages during CaOx stone formation and thereby inhibiting CaOx crystal deposition and renal tubular injury (38). In the process, the complexity of the etiology of kidney stone pathogenesis, together with the limitations of the experimental COM-induced mouse model of kidney stones raise the question of whether ADSC-EXOs might have universal applicability in treating CaOx kidney stones of all etiologies.

### 5.4 Fat graft survival rate

Fat grafting for reconstructive surgery possesses the advantages of low cost and easy accessibility, thus making it a common approach. The retention rate of fat grafts is an important measure of the success of the procedure (41). Macrophages play an important role in free oil removal, phagocytosis of dead cells and debris, and tissue inflammation. Therefore, an important link exists between macrophages and fat graft survival (41). In mouse models of fat grafting, inflammatory response reduces and survival of transplanted fat increases (41). In this process, the modulation of macrophage function and M1/M2 polarization by ADSC-EXOs plays an important role (67). The mechanism is that let-7c enriched in ADSC-EXOs downregulates the transcription factor C/EBP-δ, leading to a decrease in pro-inflammatory M1 macrophages and an increase in anti-inflammatory M2 macrophages (41). Changes in RF5, considered a key factor in M1 differentiation, were also observed experimentally: its effect on the C/EBPδ factor needs further investigation. In addition to let-7c, the impact of miR-let-7a, miR-let-7g, and miR-98 were also observed experimentally to affect the expression of C/EBP-δ; elucidating the details and mechanism of which await further future studies (41).

### 5.5 Esophageal stricture

Postoperative esophageal strictures are a major challenge following endoscopic submucosal dissection (ESD) for superficial esophageal neoplasms, with a high prevalence and limited effective treatment options (25). The main surgical treatment modalities are repeated endoscopic balloon dilatation and temporary stenting; however, these modalities can cause esophageal perforation and mediastinitis (68). Pharmacoprophylactic modalities, such as systemic administration or local injection of steroids (e.g., triamcinolone acetonide), may reduce their incidence; however, frequent use of steroids may cause adverse effects such as immunosuppression, diabetes mellitus, peptic ulcers, osteoporosis, and susceptibility to infection (69). Lai et al. demonstrated the feasibility and efficacy of MSC-EXOs for preventing esophageal strictures in a porcine ESD model (25). ADSC-EXOs contain MFGE8, for which integrin β3 is a known receptor. Activated integrin β3/SOCS3/STAT3 signaling pathway phosphorylates macrophage STAT-3, induces M2 macrophage polarization, and reduces the production of TGFβ1, playing an important role in fibrosis (25, 69). It was also observed that miR-148a-3p significantly promotes tissue angiogenesis by activating the EGFR/MAPK signaling pathway. The PI3K-Akt pathway, critically involved in cellular functions such as survival, proliferation, and migration, was the most highly enriched in the KEGG analysis. These are essential factors in mucosa treatment (25). Nonetheless, therapy requires further optimization of the dosage and duration of administration.

### 5.6 Myocardial infarction

MI is the most common disease, with acute MI being the most prevalent form (70). Acute and prolonged coronary ischemia and hypoxia can lead to myocardial necrosis and complications such as arrhythmias, aneurysms, cardiac rupture, and ultimately heart failure (71). Current treatment options include coronary artery bypass graft surgery, primary percutaneous coronary intervention, or the use of anti-remodeling drugs such as β-blockers and angiotensin-converting enzyme inhibitors (72). However, these are temporary solutions compared to heart transplantation, which is a permanent solution but has the disadvantages of a significant shortage of donor organs and the occurrence of post-transplant complications (73). Moreover, ADSCs have a promising therapeutic potential for MI (74). Several studies have reported that ADSC-EXOs exert anti-inflammatory, anti-apoptotic, pro-angiogenic, and anti-fibrotic effects, and can improve cardiac function (75, 76). In experiments applying OHA-PL hydrogel to treat a rat model of myocardial infarction, myocardial infarct area was reduced and left ventricular wall thickness was increased compared with the control group (74). In the experiment, we observed that ADSC EXOs scavenged intracellular and extracellular ROS, regulated macrophage polarization, reduced the infiltration of inflammatory cells, restored mitochondrial function, attenuated inflammation in the early stage of myocardial infarction, effectively reduced myocardial fibrosis and ventricular remodeling, promoted angiogenesis, and restored the electrophysiological function of the myocardium in the late stage of myocardial recovery. miR-125a in ADSC-EXOs, which regulates endothelial cell angiogenesis and promotes the formation of endothelial tip cells by inhibiting DLL4, is also a factor in the treatment (74). The exploration of the clinical application of the OHA-PL hydrogel is not yet complete. Its surgical application requires fundamental research and development to establish its *in situ* injection properties before this novel idea could be applied to treating other body tissues.

### 5.7 Diabetes-derived diseases

Diabetes can cause ischemia in the lower extremities leading to amputation and even death (44, 49). Chronic persistent hyperglycemia can lead to the accumulation of advanced glycation end products, tissue inflammation, and oxidative stress, triggering chronic inflammation of the vasculature and gradual destruction of blood vessels, resulting in vascular occlusion and tissue ischemia (49, 77, 78). The current clinical treatment primarily consists of pharmacological interventions and surgical hemodialysis; however, the prognosis is unsatisfactory (79). In T2DM limb ischemic mouse model, angiogenesis and blood perfusion are promoted, ADSCs significantly activate the JAK/STAT6 pathway in macrophages and induce macrophage M2 polarization (49). M2 macrophages exert anti-inflammatory effects and can initiate cellular autophagy programs to remove apoptotic cells, promote wound healing, tissue repair and regeneration, and promote angiogenesis that is important for the treatment of diabetic lower-limb ischemia (49). However, since the simple low ligation model of femoral artery in T2DM mice was used in the experiment, which is an acute process, and diabetic lower limb ischemia is a chronic process, the real efficacy still needs deeper research and demonstration. Meanwhile, it is obvious that not only the JAK/STAT6 signaling pathway and other signaling pathways are involved in macrophage M2 polarization, which needs further exploration.

## 6 Discussion

Classically and alternatively activated macrophages play important roles in tissue and cellular immune regulation. Experiments have indicated that MSCs promote M2 macrophage polarization. Follow-up studies demonstrated that MSCs induce M2 macrophage polarization via exosomes. ADSC-EXOs can regulate macrophage M1/M2 polarization and modulate tissue inflammation and immune response via the integrin β3/SOCS3/STAT3 pathway, the S1P/SK1/S1PR1 signaling pathway, and miRNAs. Possible upstream and downstream pathways as well as other mechanistic pathways merit further study (**Table 1**). An increasing number of miRNAs have been identified as playing crucial roles in the induction of macrophage polarization, suggesting that the study of these miRNAs and their upstream and downstream effects will emerge as a focal point for future research. MSC-derived exosomes do not trigger malignant transformation unlike responses observed after MSC injections. Therefore, ADSC-EXOs are expected to represent a new hope for treating challenging immune and inflammatory diseases. In recent years, research into the induction of macrophage polarization and its therapeutic applications has deepened, yielding positive results in animal experiments, such as those investigating autoimmune diseases and post-traumatic tissue repair, confirming the therapeutic and application value of ADSC-EXOs.

**Table 1:** Therapeutic mechanisms of ADSC-EXOs.

Conditions/diseases	ADSC-EXOs cargo	Mechanism	Ref
**Acute lung injury**	mtDNA	Restore macrophage mitochondrial function, inhibit TLR signaling activation.	(9)
**Bone healing**	miR-451a	Target the MIF mRNA 3’UTR, downregulate MIF expression.	(3)
**Kidney stones**	miR-23	Inhibit IRF1 expression.	(12)
**Fat graft survival rate**	let-7c	Reduce the expression of C/EBP-δ, negatively regulate TLR4	(11)
**Postoperative esophageal strictures**	MFGE8	Activate the integrin β3/SOCS3/STAT3 pathway	(29)
**Diabetic lower limb ischemia**	————	IL-4/IL-13 bind to receptors, activate the JAK/STAT6 pathway	(50)

ALI, Acute lung injury; MI, Myocardial infarction; mtDNA, Mitochondrial DNA; miR-451a, microRNA-451a; miR-23, microRNA-23; MFGE8, milk fat globule-epidermal growth factor 8; TLR, Toll-like receptor; MIF, macrophage migration inhibitory factor; IRF1, Interferon regulatory Factor 1; C/EBP-δ, CCAAT/enhancer-binding protein-δ; STAT 3, signal transducer and activator of transcription 3; IL, Interleukin.

However, ADSC-EXOs face challenges and limitations before clinical application. The current experiments were conducted in cells and animals. Further preclinical experiments must be carried out before ADSC-EXOs could be used in humans. The adaptability of exosomes to different diseases needs to be further explored, demonstrating their curative potential in inflammatory or autoimmune diseases and whether they can show corresponding weakening properties in terms of resistance. Because the current experimental animal cycle is limited to short- and long-term animal experiments, further experiments and demonstrations are needed to observe long-term side effects and safety. It has been reported that exosomes have both cancer-promoting and-suppressing effects on cancer cells; these effects need to be studied in greater depth. Simultaneously, in existing studies, there is no in-depth research on using ADSC-EXOs regarding the concentration, dose, method, and maneuverability in different diseases and the negative and positive feedback generated under such variables. Questions regarding the optimal concentration for use in the treatment of specific diseases, the relationship between the dosage and efficacy of the drug at different levels of use, and the specific requirements for the use of ADSC-EXOs owing to the characteristics of particular diseases are yet to be answered. Although several formulations have been developed, such as nano-gel particles, chitosan/gel encapsulation, and OHA-PL hydrogels in the laboratory setting, additional clinical applications require different approaches, such as fat graft survival and bone healing. Other issues, such as the production, transport, and preservation of ADSC-EXOs, will need to be considered owing to their biological and physicochemical properties.

Key factors include the effect of different sources on the final efficacy, variations in productivity among different cells, and the effect of different storage conditions on the efficacy of exosomes. Nonetheless, the powerful anti-inflammatory and immunomodulatory functions of ADSC EXOs in influencing immune, mainly macrophage cell function, provide great hope for advancing the treatment of challenging human diseases and clinical medicine.

## References

[1] Yin K Wang S Zhao RC. Exosomes from mesenchymal stem/stromal cells: a new therapeutic paradigm. biomark Res. (2019) 7:8. doi: 10.1186/s40364-019-0159-x 30992990 PMC6450000

[2] Arabpour M Saghazadeh A Rezaei N. Anti-inflammatory and M2 macrophage polarization-promoting effect of mesenchymal stem cell-derived exosomes. Int Immunopharmacol. (2021) 97:107823. doi: 10.1016/j.intimp.2021.107823 34102486

[3] Li R Li D Wang H Chen K Wang S Xu J. Exosomes from adipose-derived stem cells regulate M1/M2 macrophage phenotypic polarization to promote bone healing via miR-451a/MIF. Stem Cell Res Ther. (2022) 13:149. doi: 10.1186/s13287-022-02823-1 35395782 PMC8994256

[4] Ma ZJ Yang JJ Lu YB Liu ZY Wang XX. Mesenchymal stem cell-derived exosomes: Toward cell-free therapeutic strategies in regenerative medicine. World J Stem Cells. (2020) 12:814–40. doi: 10.4252/wjsc.v12.i8.814 PMC7477653 32952861

[5] Schorey JS Bhatnagar S. Exosome function: from tumor immunology to pathogen biology. Traffic. (2008) 9:871–81. doi: 10.1111/j.1600-0854.2008.00734.x PMC3636814 18331451

[6] Heo JS Choi Y Kim HO. Adipose-derived mesenchymal stem cells promote M2 macrophage phenotype through exosomes. Stem Cells Int. (2019) 2019:1–10. doi: 10.1155/2019/7921760 PMC6875419 31781246

[7] Lo Sicco C Reverberi D Balbi C Ulivi V Principi E Pascucci L. Mesenchymal stem cell-derived extracellular vesicles as mediators of anti-inflammatory effects: endorsement of macrophage polarization. Stem Cells Transl Med. (2017) 6:1018–28. doi: 10.1002/sctm.16-0363 PMC5442783 28186708

[8] Yao Y Xu XH Jin L. Macrophage polarization in physiological and pathological pregnancy. Front Immunol. (2019) 10:792. doi: 10.3389/fimmu.2019.00792 31037072 PMC6476302

[9] Wang J Xia J Huang R Hu Y Fan J Shu Q. Mesenchymal stem cell-derived extracellular vesicles alter disease outcomes via endorsement of macrophage polarization. Stem Cell Res Ther. (2020) 11:424. doi: 10.1186/s13287-020-01937-8 32993783 PMC7522905

[10] Harrell CR Jovicic N Djonov V Arsenijevic N Volarevic V. Mesenchymal stem cell-derived exosomes and other extracellular vesicles as new remedies in the therapy of inflammatory diseases. Cells. (2019) 8:1605. doi: 10.3390/cells8121605 31835680 PMC6952783

[11] Baharlooi H Azimi M Salehi Z Izad M. Mesenchymal stem cell-derived exosomes: A promising therapeutic ace card to address autoimmune diseases. Int J Stem Cells. (2019) 13:13–23. doi: 10.15283/ijsc19108 PMC7119210 31887849

[12] Mendt M Rezvani K Shpall E. Mesenchymal stem cell-derived exosomes for clinical use. Bone Marrow Transplant. (2019) 54:789–92. doi: 10.1038/s41409-019-0616-z 31431712

[13] Mendt M Kamerkar S Sugimoto H McAndrews KM Wu CC Gagea M. Generation and testing of clinical-grade exosomes for pancreatic cancer. JCI Insight. (2018) 3:e99263. doi: 10.1172/jci.insight.99263 29669940 PMC5931131

[14] Yu B Zhang X Li X. Exosomes derived from mesenchymal stem cells. Int J Mol Sci. (2014) 15:4142–57. doi: 10.3390/ijms15034142 PMC3975389 24608926

[15] Marote A Teixeira FG Mendes-Pinheiro B Salgado AJ. MSCs-derived exosomes: cell-secreted nanovesicles with regenerative potential. Front Pharmacol. (2016) 7:231. doi: 10.3389/fphar.2016.00231 27536241 PMC4971062

[16] Wang L Zhang S Wu H Rong X Guo J. M2b macrophage polarization and its roles in diseases. J Leukoc Biol. (2019) 106:345–58. doi: 10.1002/JLB.3RU1018-378RR PMC7379745 30576000

[17] Li J Xue H Li T Chu X Xin D Xiong Y. Exosomes derived from mesenchymal stem cells attenuate the progression of atherosclerosis in ApoE–/- mice via miR-let7 mediated infiltration and polarization of M2 macrophage. Biochem Biophys Res Commun. (2019) 510:565–72. doi: 10.1016/j.bbrc.2019.02.005 30739785

[18] Sica A Mantovani A. Macrophage plasticity and polarization: *in vivo* veritas. J Clin Invest. (2012) 122:787–95. doi: 10.1172/JCI59643 PMC3287223 22378047

[19] Martinez FO Gordon S Locati M Mantovani A. Transcriptional profiling of the human monocyte-to-macrophage differentiation and polarization: new molecules and patterns of gene expression1. J Immunol. (2006) 177:7303–11. doi: 10.4049/jimmunol.177.10.7303 17082649

[20] O’Shea JJ Paul WE. Mechanisms underlying lineage commitment and plasticity of helper CD4+ T cells. Science. (2010) 327:1098–102. doi: 10.1126/science.1178334 PMC2997673 20185720

[21] Lampiasi N Russo R Zito F. The alternative faces of macrophage generate osteoclasts. BioMed Res Int. (2016) 2016:9089610. doi: 10.1155/2016/9089610 26977415 PMC4761668

[22] Xu R Zhang F Chai R Zhou W Hu M Liu B. Exosomes derived from pro-inflammatory bone marrow-derived mesenchymal stem cells reduce inflammation and myocardial injury via mediating macrophage polarization. J Cell Mol Med. (2019) 23:7617–31. doi: 10.1111/jcmm.v23.11 PMC6815833 31557396

[23] Wu J. Knockdown of milk-fat globule EGF factor-8 suppresses glioma progression in GL261 glioma cells by repressing microglial M2 polarization. J Cell Physiol. (2020) 235(11):8679–90. doi: 10.1002/jcp.29712 32324268

[24] Brissette MJ Lepage S Lamonde AS Sirois I Groleau J Laurin LP. MFG-E8 released by apoptotic endothelial cells triggers anti-inflammatory macrophage reprogramming. PloS One. (2012) 7:e36368. doi: 10.1371/journal.pone.0036368 22558449 PMC3340380

[25] Lai H Yip HC Gong Y Chan KF Leung KKC Chan MS. MFGE8 in exosomes derived from mesenchymal stem cells prevents esophageal stricture after endoscopic submucosal dissection in pigs. J Nanobiotechnology. (2024) 22:143. doi: 10.1186/s12951-024-02429-0 38561800 PMC10986023

[26] Chen L Qu J Cheng T Chen X Xiang C. Menstrual blood-derived stem cells: toward therapeutic mechanisms, novel strategies, and future perspectives in the treatment of diseases. Stem Cell Res Ther. (2019) 10:406. doi: 10.1186/s13287-019-1503-7 31864423 PMC6925480

[27] Yuan Y Ni S Zhuge A Li L Li B. Adipose-derived mesenchymal stem cells reprogram M1 macrophage metabolism via PHD2/HIF-1α Pathway in colitis mice. Front Immunol. (2022) 13:859806. doi: 10.3389/fimmu.2022.859806 35757749 PMC9226317

[28] Yang CY Chang PY Chen JY Wu BS Yang AH Lee OKS. Adipose-derived mesenchymal stem cells attenuate dialysis-induced peritoneal fibrosis by modulating macrophage polarization via interleukin-6. Stem Cell Res Ther. (2021) 12:193. doi: 10.1186/s13287-021-02270-4 33741073 PMC7977319

[29] Xie Z Hao H Tong C Cheng Y Liu J Pang Y. Human umbilical cord-derived mesenchymal stem cells elicit macrophages into an anti-inflammatory phenotype to alleviate insulin resistance in type 2 diabetic rats. Stem Cells. (2016) 34:627–39. doi: 10.1002/stem.2238 26523620

[30] Mauer J Chaurasia B Goldau J Vogt MC Ruud J Nguyen KD. Interleukin-6 signaling promotes alternative macrophage activation to limit obesity-associated insulin resistance and endotoxemia. Nat Immunol. (2014) 15:423–30. doi: 10.1038/ni.2865 PMC4161471 24681566

[31] Du L Lin L Li Q Liu K Huang Y Wang X. IGF-2 preprograms maturing macrophages to acquire oxidative phosphorylation-dependent anti-inflammatory properties. Cell Metab. (2019) 29:1363–1375.e8. doi: 10.1016/j.cmet.2019.01.006 30745181

[32] Hsin JP Lu Y Loeb GB Leslie CS Rudensky AY. The effect of cellular context on miR-155-mediated gene regulation in four major immune cell types. Nat Immunol. (2018) 19:1137–45. doi: 10.1038/s41590-018-0208-x PMC6158091 30224821

[33] Cronk JC Derecki NC Ji E Xu Y Lampano AE Smirnov I. Methyl-CpG binding protein 2 regulates microglia and macrophage gene expression in response to inflammatory stimuli. Immunity. (2015) 42:679–91. doi: 10.1016/j.immuni.2015.03.013 PMC4407145 25902482

[34] Mirzaei H. Stroke in women: risk factors and clinical biomarkers. J Cell Biochem. (2017) 118:4191–202. doi: 10.1002/jcb.v118.12 28498508

[35] Liu XS Chopp M Zhang RL Zhang ZG. MicroRNAs in cerebral ischemia-induced neurogenesis. J Neuropathol Exp Neurol. (2013) 72:718–22. doi: 10.1097/NEN.0b013e31829e4963 PMC3746334 23860031

[36] Graham A Falcone T Nothnick WB. The expression of microRNA-451 in human endometriotic lesions is inversely related to that of macrophage migration inhibitory factor (MIF) and regulates MIF expression and modulation of epithelial cell survival. Hum Reprod Oxf Engl. (2015) 30:642–52. doi: 10.1093/humrep/dev005 PMC4325675 25637622

[37] Li Q Li Y Zhang D Gao H Gao X. Downregulation of microRNA-451 improves cell migration, invasion and tube formation in hypoxia-treated HUVECs by targeting MIF. Mol Med Rep. (2019) 20:1167–77. doi: 10.3892/mmr.2019.10357 PMC6625462 31173234

[38] Yifan Z Shengli Z Min W Wenjie C Yi S Luwei X. Exosomes from miR-23 overexpressing stromal cells suppress M1 macrophage and inhibit calcium oxalate deposition in hyperoxaluria rat model. BioMed Res Int. (2023) 2023:1–10. doi: 10.1155/2023/2883623 PMC10667050 38027040

[39] Jiang M Wang H Jin M Yang X Ji H Jiang Y. Exosomes from miR-30d-5p-ADSCs reverse acute ischemic stroke-induced, autophagy-mediated brain injury by promoting M2 microglial/macrophage polarization. Cell Physiol Biochem. (2018) 47:864–78. doi: 10.1159/000490078 29807362

[40] Banerjee S Xie N Cui H Tan Z Yang S Icyuz M. MicroRNA let-7c regulates macrophage polarization. J Immunol. (2013) 190:6542–9. doi: 10.4049/jimmunol.1202496 PMC3679284 23667114

[41] Hao X Guo Y Wang R Yu X He L Shu M. Exosomes from adipose-derived mesenchymal stem cells promote survival of fat grafts by regulating macrophage polarization via let-7c. Acta Biochim Biophys Sin. (2021) 53:501–10. doi: 10.1093/abbs/gmab018 33704368

[42] He W Xu C Huang Y Zhang Q Chen W Zhao C. Therapeutic potential of ADSC-EV-derived lncRNA DLEU2: A novel molecular pathway in alleviating sepsis-induced lung injury via the miR-106a-5p/LXN axis. Int Immunopharmacol. (2024) 130:111519. doi: 10.1016/j.intimp.2024.111519 38442573

[43] Wang F Jiang M Chi Y Huang G Jin M. Exosomes from circRNA-Ptpn4 can modify ADSC treatment and repair nerve damage caused by cerebral infarction by shifting microglial M1/M2 polarization. Mol Cell Biochem. (2024) 479(8):2081–92. doi: 10.1007/s11010-023-04824-x 37632638

[44] Yin D Shen G. Exosomes from adipose-derived stem cells regulate macrophage polarization and accelerate diabetic wound healing via the circ-Rps5/miR-124-3p axis. Immun Inflammation Dis. (2024) 12:e1274. doi: 10.1002/iid3.v12.6 PMC11184652 38888351

[45] Shang Y Sun Y Xu J Ge X Hu Z Xiao J. Exosomes from mmu_circ_0001359-modified ADSCs attenuate airway remodeling by enhancing foxO1 signaling-mediated M2-like macrophage activation. Mol Ther - Nucleic Acids. (2020) 19:951–60. doi: 10.1016/j.omtn.2019.10.049 PMC6997502 32018116

[46] Xia L Zhang C Lv N Liang Z Ma T Cheng H. AdMSC-derived exosomes alleviate acute lung injury via transferring mitochondrial component to improve homeostasis of alveolar macrophages. Theranostics. (2022) 12:2928–47. doi: 10.7150/thno.69533 PMC8965475 35401830

[47] Yang S Li F Lu S Ren L Bian S Liu M. Ginseng root extract attenuates inflammation by inhibiting the MAPK/NF-κB signaling pathway and activating autophagy and p62-Nrf2-Keap1 signaling *in vitro* and in *vivo* . J Ethnopharmacol. (2022) 283:114739. doi: 10.1016/j.jep.2021.114739 34648903

[48] Wang Y Wang GZ Rabinovitch PS Tabas I. Macrophage mitochondrial oxidative stress promotes atherosclerosis and NF-κB-mediated inflammation in macrophages. Circ Res. (2014) 114:421–33. doi: 10.1161/CIRCRESAHA.114.302153 PMC3946745 24297735

[49] Wang X Chen S Lu R Sun Y Song T Nie Z. Adipose-derived stem cell-secreted exosomes enhance angiogenesis by promoting macrophage M2 polarization in type 2 diabetic mice with limb ischemia via the JAK/STAT6 pathway. Heliyon. (2022) 8:e11495. doi: 10.1016/j.heliyon.2022.e11495 36406687 PMC9668683

[50] Gordon S Martinez FO. Alternative activation of macrophages: mechanism and functions. Immunity. (2010) 32:593–604. doi: 10.1016/j.immuni.2010.05.007 20510870

[51] Zhou D Huang C Lin Z Zhan S Kong L Fang C. Macrophage polarization and function with emphasis on the evolving roles of coordinated regulation of cellular signaling pathways. Cell Signal. (2014) 26:192–7. doi: 10.1016/j.cellsig.2013.11.004 24219909

[52] Deng S Zhou X Ge Z Song Y Wang H Liu X. Exosomes from adipose-derived mesenchymal stem cells ameliorate cardiac damage after myocardial infarction by activating S1P/SK1/S1PR1 signaling and promoting macrophage M2 polarization. Int J Biochem Cell Biol. (2019) 114:105564. doi: 10.1016/j.biocel.2019.105564 31276786

[53] Zhao H Shang Q Pan Z Bai Y Li Z Zhang H. Exosomes from adipose-derived stem cells attenuate adipose inflammation and obesity through polarizing M2 macrophages and beiging in white adipose tissue. Diabetes. (2018) 67:235–47. doi: 10.2337/db17-0356 29133512

[54] Sapoznikov A Gal Y Falach R Sagi I Ehrlich S Lerer E. Early disruption of the alveolar-capillary barrier in a ricin-induced ARDS mouse model: neutrophil-dependent and -independent impairment of junction proteins. Am J Physiol-Lung Cell Mol Physiol. (2019) 316:L255–68. doi: 10.1152/ajplung.00300.2018 30382767

[55] Hayes M Curley G Ansari B Laffey JG. Clinical review: Stem cell therapies for acute lung injury/acute respiratory distress syndrome - hope or hype? Crit Care. (2012) 16:205. doi: 10.1186/cc10570 22424108 PMC3681334

[56] Levitt JE Calfee CS Goldstein BA Vojnik R Matthay MA. Early acute lung injury: Criteria for identifying lung Injury prior to the need for positive pressure ventilation. Crit Care Med. (2013) 41:1929–37. doi: 10.1097/CCM.0b013e31828a3d99 PMC3748809 23782966

[57] Yu W-w Lu Z Zhang H Kang Y-h Mao Y Wang H-h. The anti-inflammatory and protective property of daphnetin in the endotoxin-induced lung injury. J Agric Food Chem. (2014) 62(51):12315–25. doi: 10.1021/jf503667v 25419854

[58] Baldwin P Li DJ Auston DA Mir HS Yoon RS Koval KJ. Autograft, allograft, and bone graft substitutes: clinical evidence and indications for use in the setting of orthopaedic trauma surgery. J Orthop Trauma. (2019) 33:203–13. doi: 10.1097/BOT.0000000000001420 30633080

[59] Likhterov I Roche AM Urken ML. Contemporary osseous reconstruction of the mandible and the maxilla. Oral Maxillofac Surg Clin N Am. (2019) 31:101–16. doi: 10.1016/j.coms.2018.08.005 30449523

[60] Shegarfi H Reikeras O. Review article: bone transplantation and immune response. J Orthop Surg. (2009) 17:206–11. doi: 10.1177/230949900901700218 19721154

[61] Agarwal R García AJ. Biomaterial strategies for engineering implants for enhanced osseointegration and bone repair. Adv Drug Delivery Rev. (2015) 94:53–62. doi: 10.1016/j.addr.2015.03.013 PMC4598264 25861724

[62] Kim HD Amirthalingam S Kim SL Lee SS Rangasamy J Hwang NS. Biomimetic materials and fabrication approaches for bone tissue engineering. Adv Healthc Mater. (2017) 6(23). doi: 10.1002/adhm.201700612 29171714

[63] D’Costa MR Haley WE Mara KC Enders FT Vrtiska TJ Pais VM. Symptomatic and radiographic manifestations of kidney stone recurrence and their prediction by risk factors: A prospective cohort study. J Am Soc Nephrol JASN. (2019) 30:1251–60. doi: 10.1681/ASN.2018121241 PMC6622409 31175141

[64] Li C Lu Y. Resveratrol attenuates oxalate-induced renal oxidative injury and calcium oxalate crystal deposition by regulating TFEB-induced autophagy pathway. Front Cell Dev Biol. (2021) 9. doi: 10.3389/fcell.2021.638759 PMC7947311 33718378

[65] Taguchi K Okada A Unno R Hamamoto S Yasui T. Macrophage function in calcium oxalate kidney stone formation: A systematic review of literature. Front Immunol. (2021) 12:673690. doi: 10.3389/fimmu.2021.673690 34108970 PMC8182056

[66] Thongboonkerd V. Roles of macrophage exosomes in immune response to calcium oxalate monohydrate crystals. Front Immunol. (2018) 9. doi: 10.3389/fimmu.2018.00316 PMC5835051 29535716

[67] Helwa I Cai J Drewry MD Zimmerman A Dinkins MB Khaled ML. A comparative study of serum exosome isolation using differential ultracentrifugation and three commercial reagents. PloS One. (2017) 12:e0170628. doi: 10.1371/journal.pone.0170628 28114422 PMC5256994

[68] Shibagaki K. Esophageal triamcinolone acetonide-filling method: a novel procedure to prevent stenosis after extensive esophageal endoscopic submucosal dissection (with videos). Gastrointest Endosc. (2017) 87(2):380–9. doi: 10.1016/j.gie.2017.08.016 28843584

[69] Yamaguchi N Isomoto H Nakayama T Hayashi T Nishiyama H Ohnita K. Usefulness of oral prednisolone in the treatment of esophageal stricture after endoscopic submucosal dissection for superficial esophageal squamous cell carcinoma. Gastrointest Endosc. (2011) 73(6):1115–21. doi: 10.1016/j.gie.2011.02.005 21492854

[70] Pham TP Boon RA. Exosomes and non-coding RNA, the healers of the heart? Cardiovasc Res. (2019) 116(2):258–9. doi: 10.1093/cvr/cvz190 31321404

[71] Lin B Chen X Lu C Xu J Qiu Y Liu X. Loss of exosomal LncRNA HCG15 prevents acute myocardial ischemic injury through the NF-κB/p65 and p38 pathways. Cell Death Dis. (2021) 12:1007. doi: 10.1038/s41419-021-04281-8 34707098 PMC8551195

[72] Sun SJ Wei R Li F Liao SY Tse HF. Mesenchymal stromal cell-derived exosomes in cardiac regeneration and repair. Stem Cell Rep. (2021) 16:1662–73. doi: 10.1016/j.stemcr.2021.05.003 PMC8282428 34115984

[73] Sharma V Dash SK Govarthanan K Gahtori R Negi N Barani M. Recent advances in cardiac tissue engineering for the management of myocardium infarction. Cells. (2021) 10:2538. doi: 10.3390/cells10102538 34685518 PMC8533887

[74] Ren Y Wang W Yu C Wang Y Qiu Y Yue Z. An injectable exosome-loaded hyaluronic acid-polylysine hydrogel for cardiac repair via modulating oxidative stress and the inflammatory microenvironment. Int J Biol Macromol. (2024) 275(Pt 2):133622. doi: 10.1016/j.ijbiomac.2024.133622 38969034

[75] Mondal J Pillarisetti S Junnuthula V Saha M Hwang SR Park I-k. Hybrid exosomes, exosome-like nanovesicles and engineered exosomes for therapeutic applications. J Controlled Release. (2023) 353:1127–49. doi: 10.1016/j.jconrel.2022.12.027 36528193

[76] Das A Nikhil A Shiekh PA Yadav B Jagavelu K Kumar A. Ameliorating impaired cardiac function in myocardial infarction using exosome-loaded gallic-acid-containing polyurethane scaffolds. Bioact Mater. (2023) 33:324–40. doi: 10.1016/j.bioactmat.2023.11.009 PMC10701288 38076649

[77] Yang P Feng J Peng Q Liu X Fan Z. Advanced glycation end products: potential mechanism and therapeutic target in cardiovascular complications under diabetes. Oxid Med Cell Longev. (2019) 2019:9570616. doi: 10.1155/2019/9570616 31885827 PMC6925928

[78] Groener JB Oikonomou D Cheko R Kender Z Zemva J Kihm L. Methylglyoxal and advanced glycation end products in patients with diabetes – what we know so far and the missing links. Exp Clin Endocrinol Diabetes. (2019) 127:497–504. doi: 10.1055/s-0043-106443 28407670

[79] Mills JL Conte MS Armstrong DG Pomposelli FB Schanzer A Sidawy AN. The Society for Vascular Surgery Lower Extremity Threatened Limb Classification System: Risk stratification based on Wound, Ischemia, and foot Infection (WIfI). J Vasc Surg. (2014) 59:220–234.e2. doi: 10.1016/j.jvs.2013.08.003 24126108

